# Hydrogen Peroxide Treatment of Softwood-Derived Poly(Ethylene Glycol)-Modified Glycol Lignin at Room Temperature

**DOI:** 10.3390/molecules28041542

**Published:** 2023-02-05

**Authors:** Thi Thi Nge, Yuki Tobimatsu, Shiho Takahashi, Toshiaki Umezawa, Tatsuhiko Yamada

**Affiliations:** 1 Center for Advanced Materials, Forestry and Forest Products Research Institute (FFPRI), 1 Matsunosato, Tsukuba 305-8687, Japan; 2 Research Institute for Sustainable Humanosphere, Kyoto University, Gokasho, Uji, Kyoto 611-0011, Japan; 3 Department of Forest Resources Chemistry, Forestry and Forest Products Research Institute (FFPRI), 1 Matsunosato, Tsukuba 305-8687, Japan

**Keywords:** glycol lignin, poly(ethylene) glycol solvolysis, hydrogen peroxide, oxidative depolymerization, carboxy functionalization

## Abstract

Recently, a large-scale production system of softwood-derived poly(ethylene glycol) (PEG)-modified glycol lignin (GL) was developed to produce high-quality lignin derivatives with substantially controlled chemical structures and attractive thermal properties. In this study, the further upgrading of GL properties with carboxy functionalization was demonstrated through the room-temperature hydrogen peroxide (H_2_O_2_) treatment with the mass ratio of H_2_O_2_ to GL, 1:1 and 1:3, for 7 d. The changes in the chemical structure, carboxy group content, molecular weight, and thermal properties of the insoluble portions of partially oxidized glycol lignins (OGLs) were then investigated. Nuclear magnetic resonance and thioacidolysis data revealed that the oxidative functionalization involved the cleavage of *β*–*O*–4 linkages and the oxidative cleavage of guaiacyl aromatic rings into muconic acid-type structures. This was validated by attenuated total reflectance-Fourier transform infrared (ATR-FTIR) spectroscopy and potentiometric titration. Overall, the results suggested that the varying outcomes of carboxy group content (0.81–2.04 mmol/g OGL) after 7-d treatment depended on the type of the GL origin having varying amounts of the retained native lignin structure (e.g., *β*–*O*–4 linkages), which were prepared from different source-wood-meal sizes and PEG molecular masses.

## 1. Introduction

In current biorefinery processes, such as pulp and paper manufacturing processes, various technologies have been developed to produce high-quality pulp from lignocellulosic biomass. Meanwhile, lignin from the remainder of the biomass ends up as a waste/byproduct and has mainly been used as an internal energy source to recover chemicals used in the pulping processes [1]. Lignin contributes up to 30% of plant cell walls [2] and is the most abundant, renewable, and sustainable source of aromatics. Thus, lignin valorization and targeted functionalization within biorefinery processes should be further explored and developed [3]. Lignin valorization includes (1) the use of lignin as a macromolecule for developing biobased substitutes for fossil-based phenolic polymer precursors [4,5] and (2) the oxidative depolymerization/functionalization of lignin into low-molecular-weight fragments, phenolic or other aromatic compounds, and monocarboxylic and dicarboxylic acids, which are important platform chemicals for the polymer, pharmaceutical, and food industries [6,7]. However, the heterogeneity and impurity of industrial lignin, such as kraft lignin, limit its industrial implementation for large-scale production. Hence, some successful technologies have been developed to solve these issues. For example, the LignoBoost process, which is integrated into the chemical pulping process, has been realized as a large-scale plant production process [8].

Recently, an alternative biorefinery process has been developed to produce high-quality lignin derivatives from softwood biomass through an acid-catalyzed solvolysis process in a large-scale batch reactor (50 kg wood meal per batch) using poly(ethylene glycol) (PEG) as a solvolysis reagent [9]. As a high boiling solvent, the PEG solvolysis system enables the operation of the large-scale process under atmospheric pressure. In addition, PEG serves as a grafting reagent that is incorporated into isolated lignin fragments during solvolysis. As softwood lignins mainly comprise guaiacyl (G) units, using softwood meals (Japanese cedar, JC) as an initial biomass resource enables the system to produce PEG-modified lignin derivatives, namely glycol lignins (GLs), with reduced structural heterogeneity in terms of aromatic units. A two-dimensional (2D) nuclear magnetic resonance (NMR) analysis confirmed the chemical structure of PEG-modified GLs, where all *α*-OH-*β*–*O*–4 units in the source JC lignins were reacted during PEG solvolysis, producing GLs containing *α*-PEG-*β*–*O*–4 units and intact *β*–5 and *β*–*β* linkage units [9]. Lignin PEGylation contributed to the enhanced thermal flow of GLs, [9,10,11] which is important for fabricating melt-processed materials. Consequently, the use of GL as a green material in fabricating flexible electronic substrates [12], elastomeric epoxy resins [13], heat-resistant films [14], and fiber-reinforced plastics (FRPs) [11] was explored to extensively apply GLs in developing sustainable industrialization.

Simultaneously, research on further upgrading GL properties is continuously expanding the potential industrial applications of GLs. For example, upgrading GL with carboxy functionalization is covered in this study. Carboxy groups can be introduced by the hydrogen peroxide (H_2_O_2_) treatment of lignin under alkaline [15,16,17] or acidic media [15,18,19,20] without a metal catalyst at room temperature [19,20] or at a certain elevated temperature [15,16,17,18]. H_2_O_2_ is a safe and clean oxidizing agent and produces only water as its byproduct through oxidation. As a green oxidant, H_2_O_2_ is an industrially attractive and environmentally friendly chemical commonly used as one of the bleaching reagents in the pulp and paper industry. Aside from reacting with chromophoric groups, it can also degrade and solubilize the low-molecular-weight reactive lignin fragments. Lignin H_2_O_2_ oxidation using lignin model compounds and technical lignins has been extensively studied to understand the lignin oxidation process suitable for the efficient production of green end products [such as aromatic acids, aldehydes, phenolic compounds, and monocarboxylic and dicarboxylic acids (DCAs)] as renewable replacements for fossil-based building blocks in polymer and material syntheses [21,22,23,24,25,26,27,28,29]. Over the past decades, the application of H_2_O_2_ as an oxidizing agent for the conversion of biomass to chemicals has been a significant industrial interest consistent with the growing awareness for the development of a sustainable environment [30].

As an oxidant, H_2_O_2_ can act as an electrophilic or nucleophilic agent, depending on the pH of the reaction medium. In an acidic medium, H_2_O_2_ acts as an electrophile by forming hydroxyl cations (HO^+^), whereas it acts as a nucleophile by forming hydroperoxide anions (HOO^−^) under alkaline conditions, as shown in Equations (1) and (2), respectively [15].

H_2_O_2_ + H^+^ ⇋ H_3_O_2_^+^ ⇋ H_2_O + HO^+^ (1)

H_2_O_2_ + HO^−^ ⇋ H_2_O + HOO^−^ (2)

Numerous studies exist on the oxidative depolymerization of lignin and lignin model compounds through H_2_O_2_ treatment under alkaline conditions; however, there are limited studies on the mechanism of the breakdown of lignin by H_2_O_2_ under acidic conditions have been reported [15,21]. It has been proposed that the reactions responsible for this process are similar to those observed in the peracetic acid depolymerization of lignin and lignin model compounds, where a strong electrophilic species (hydroxyl cation) produced by the heterolytic cleavage of a peroxidic bond readily reacts with electron-rich sites in lignin, including aromatic ring and olefinic side-chain structures. Aromatic ring hydroxylation, oxidative demethylation, oxidative ring cleavage, side-chain displacement, β-aryl ether bond cleavage, and olefin structure epoxidation proceed depending on the peroxide structure and the reaction conditions employed [21,31,32].

GLs were produced by the acidification (3N H_2_SO_4_) of alkaline JC/PEG solvolysis liquor, followed by successive washing with water, which had a pH of 4.5–5. Accordingly, the reaction mixtures of H_2_O_2_/GLs were considered to be acidic after mixing with an aqueous H_2_O_2_ solution. Therefore, the H_2_O_2_-mediated oxidation of GLs was supposed to occur under acidic conditions, where electrophilic hydroxyl cations (HO^+^) attacked the electron-rich sites of the lignin structural units. Alternatively, reactions involving hydroxyl radicals generated by the decomposition of H_2_O_2_ are also possible, although generations of hydroxyl radicals from H_2_O_2_ at an ambient temperature typically require proper metal catalysts [27]. Since this study focuses on upgrading GL properties with carboxy functionalization through H_2_O_2_ treatment, insoluble oxidized glycol lignin (OGL) is preferred to soluble OGL. Therefore, the H_2_O_2_ treatment of GL was conducted by simple mixing with an aqueous H_2_O_2_ solution without additional catalysts, and the oxidation reaction was monitored at room temperature. The structural changes and carboxy groups of OGLs induced by the H_2_O_2_ treatment were investigated using 2D heteronuclear single quantum coherence (HSQC) NMR, thioacidolysis, and attenuated total reflectance-Fourier transform infrared (ATR-FTIR) analyses, as well as potentiometric titration, to gain insight into carboxy functionalization under mild reaction conditions. Consequently, a correlation between the values of the qualitative ATR-FTIR and the quantitative potentiometric titration analyses was established for a simple determination of the carboxy group content of OGLs. Additionally, changes in the molecular weight distribution and thermal properties of OGLs due to oxidative functionalization were determined.

## 2. Results and Discussion

GLs used in this study were prepared directly from the acid-catalyzed PEG solvolysis of softwood meal (JC) at 140 °C for 90 min using three size distributions [average particle size: JC-L (1.6 mm) > JC-M (0.8 mm) > JC-S (0.4 mm)] and three types of liquid PEG (molecular mass: PEG200 < PEG400 < PEG600). As reported in our previous study, the chemical structure, molecular mass, and thermal properties of GLs varied depending on the JC size and PEG molecular mass. Reducing the JC size and PEG molecular mass enhanced the increased reactivity of the JC particle substrates and PEG in the solvolysis reactions. This promoted lignin PEGylation and further acid-induced chemical rearrangements of GLs as demonstrated by 2D NMR and thioacidolysis, eventually leading to enhanced lignin yield. The reduction of the three major inter-monomeric linkage signals (*α*-PEG-*β*–*O*–4, *β*–5, and *β*–*β* linkages) and the amount of *β*–*O*–4 units as well as the molecular weight of the GL obtained with the decrease in JC size and an increase in PEG molecular mass [9]. Thus, varying degrees of oxidation reactions among GLs are expected to occur in this study. Forty partially oxidized glycol lignins (OGLs) (Table 1) were obtained through H_2_O_2_-mediated oxidation treatment at room temperature (25 °C–27 °C) for 1, 3, 5, and 7 d. The OGLs were denoted as OGLxxxY11-z and OGLxxxY13-z, where xxx represents the molecular mass of PEG (200, 400, 600); Y represents the source-wood-meal sizes (L, M, S); z represents the H_2_O_2_ treatment time (1, 3, 5, and 7 d); and 11 and 13 represent the H_2_O_2_/GL mass ratios, 1:1 and 1:3, respectively (Table 1).

**Figure 1:**
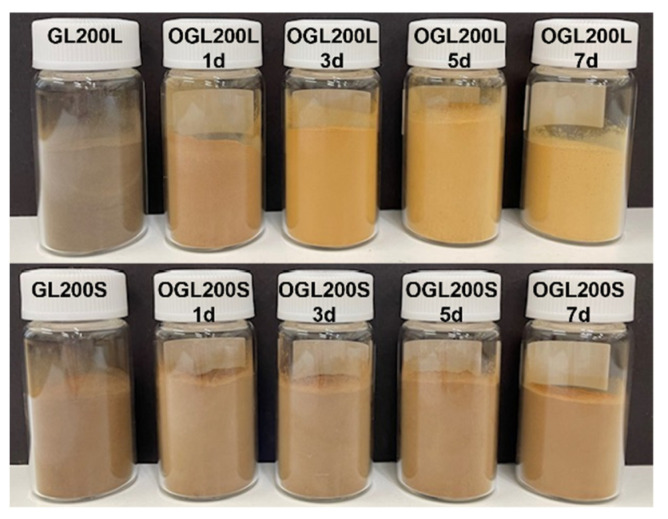
Illustration of OGLs (OGL200L11 and OGL200S11 series) after room-temperature H_2_O_2_-mediated oxidation treatment (GL200L and GL200S were prepared from the PEG200 solvolysis of JC wood meal L and S, respectively).

The mild H_2_O_2_-mediated oxidation treatment of the five original GLs resulted in OGLs with varying degrees of carboxy functionalization, as indicated by the visual color changes of the recovered OGLs with an increase in treatment time (Figure 1). The color changes in the OGL200L series become more pronounced with an increase in treatment time than those of the OGL200S series under the same H_2_O_2_-mediated oxidation treatment. The product yields, molecular mass, chemical structure, carboxy functionalization, and thermal properties of OGLs are discussed based on two primary raw materials—JC sizes and PEGs—used to prepare the original GLs (Table 1).

### 2.1. Oxidized Glycol Lignin Yields

**Figure 2:**
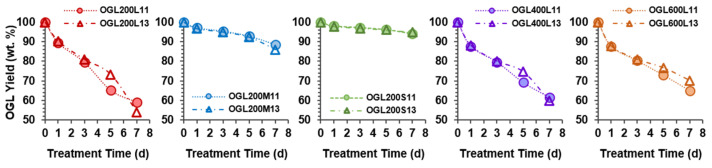
OGLs yields as a function of H_2_O_2_ treatment time.

Similar OGLs yields were observed for the 1-d and 3-d treatment times in both H_2_O_2_/GL ratios (1:1 and 1:3) (Figure 2). A slight change was observed for the OGLs obtained from the 5-d and 7-d treatment times, which was not consistent in all the OGLs series. As indicated by the product yield as a function of treatment time in Figure 2, the oxidation induced the partial degradation of GL as a soluble low-molecular-weight fragment in the reaction slurry. The decrease in OGLs yields with an increase in treatment time significantly depended on the original GLs prepared from three source-wood-meal sizes in the following order: the product yields of OGL200L << (OGL200M < OGL200S) series.

Meanwhile, the product yields of OGLs obtained from reacting control GLs with various PEG molecular masses showed a slight difference in value, particularly for the 5-d and 7-d samples, in the following order: OGL200L < OGL400L < OGL600L series. Accordingly, maximum and minimum mass losses of 45% and 5% were observed for the OGL200L13-7d and OGL200S11/13-7d samples, respectively. The superior reactivity of GLxxxL (GL200L, GL400L, and GL600L) compared to GL200M and GL200S suggested that the macromolecular structure of GLxxxL was more susceptible to H_2_O_2_-mediated oxidation than the other two GL series, even under ambient conditions. In addition, the GL/H_2_O_2_ mass ratio of 1:1 was suitable for the oxidation treatment of all original GLs used in this study.

Klason lignin analysis (Table 2) was performed to examine the effect of the H_2_O_2_ treatment. The Klason lignin content increased slightly in the 1-d and/or 3-d samples, followed by a gradual decrease in the 5-d and 7-d samples for OGL200L, OGL400L, OGL600L, and OGL200M. However, the OGL200S samples showed a similar value regardless of the treatment time. A notable increase in acid-soluble lignin content with an increase in treatment time was observed in all series. This increase was higher in the OGL200L, OGL400L, and OGL600L series than in the OGL200M and OGL200S series. Consequently, the total lignin content (i.e., the sum of the Klason lignin and acid-soluble lignin content) remained nearly the same, except in the OGLxxxL-7d samples. This result further suggested that (1) an effective oxidation reaction occurred in the OGLxxxL series, and (2) the structure of OGLs produced by accelerated oxidation with a prolonged treatment time has increased susceptibility to acid hydrolysis during Klason lignin analysis.

### 2.2. Molecular Weight Distribution of Oxidized Glycol Lignins

**Figure 3:**
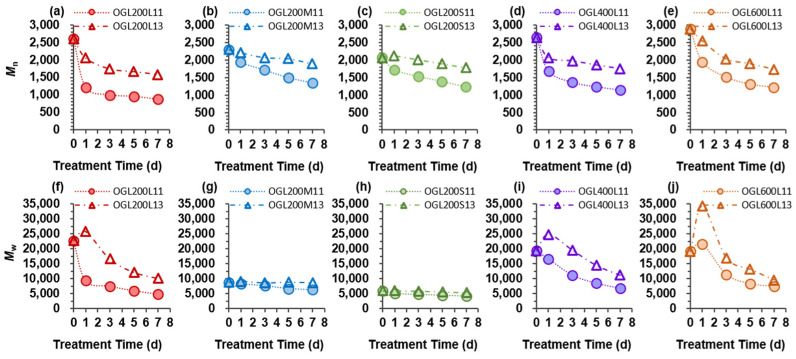
Molecular weight, *M*_n_ (**a**–**e**) and *M*_w_ (**f**–**j**) of OGLs as a function of H_2_O_2_ treatment time. (**a**,**f**) OGL200L series, (**b**,**g**) OGL200M series, (**c**,**h**) OGL200S series, (**d**,**i**) OGL400L series, and (**e**,**j**) OGL600L series were obtained through the room-temperature H_2_O_2_ treatment of GLs with H_2_O_2_/GL 1:1 and 1:3 mass ratios.

After room-temperature H_2_O_2_ treatment, the large-molecular-weight fractions remained in a solid state, while the low-molecular-weight fragments generated were dissolved in the aqueous H_2_O_2_ solution. The extent of mass loss was in the following order: OGL200L > OGL400L > OGL600L > OGL200M > OGL200S (Figure 2). Notably, the weight average molecular weight (*M*_w_) and the number average molecular weight (*M*_n_) of solid OGLs obtained from the H_2_O_2_/GL-1:1 treatment were lower than those obtained from the H_2_O_2_/GL-1:3 treatments (Figure 3). Generally, the *M*_n_ of solid OGLs obtained from both treatment ratios decreased with an increase in treatment time. After the 7-d treatment, the *M*_n_ was reduced for the 1:1 and 1:3 mass ratios in the following order: OGL200L (67%) > OGL400L, OGL600L (58%) > OGL200M (41%) > OGL200S (40%) and OGL200L, OGL600L (40%) > OGL400L (34%) > OGL200M (18%) > OGL200S (14%) (Figure 3a–e).

A particular effect between the two treatment ratios was observed in the *M*_w_ of OGLxxxL-1d samples. The *M*_w_ of the OGL200L13, OGL400L13, and OGL600L13 samples increased after the 1-d treatment, followed by a gradual decrease with the increasing treatment time. In addition, the *M*_w_ of OGLxxxL11 exhibited three different decreasing trends depending on the PEG molecular mass incorporated with the control GL: (1) a dramatic decrease after the 1-d treatment, followed by a slightly decreasing trend in the OGL200L11 series; (2) a gradually decreasing trend in the OGL400L11 series; and (3) a slight increase after the 1-d treatment, followed by a gradually decreasing trend in the OGL600L11 series. Conversely, the *M*_w_ of the OGL200M and OGL200S series from both mass ratios exhibited a slightly decreasing trend with an increase in treatment time. After 7-d treatment, the *M*_w_ was reduced in the following order: OGL200L (79%) > OGL400L (65%) > OGL600L (62%) > OGL200M, OGL200S (30%) and OGL200L (56%) > OGL600L (50%) > OGL400L (43%) > OGL200M (10%) > OGL200S (2%), for 1:1 and 1:3 mass ratios, respectively (Figure 3f–j).

The lower *M*_w_ and *M*_n_ values in the 1:1 mass ratio compared to the 1:3 mass ratio, despite no significant difference in OGLs yields, suggested that the H_2_O_2_-mediated oxidative treatment of the 1:1 mass ratio was suitable for effective GL depolymerization at ambient temperatures. At a high mass load of H_2_O_2_ (H_2_O_2_/GL, 1:3), oxidation likely proceeds rapidly initially (1-d treatment), resulting in structural rearrangements, including GL depolymerization and partial liberation of PEG moieties, which are competitive with the condensation or recombination of the intermediates. The results in Figure 3 show that a sudden increase in the *M*_w_ was initially observed in OGLxxxL13 1-d samples before proceeding to gradual depolymerization with the time extension, particularly in GLs incorporated with long PEG chain lengths (GL600L > GL400L > GL200L). This sudden increase in the *M*_w_ of GLxxxL13 1-d samples implied that condensation reactions and/or recombination of intermediates from GL proceeded before depolymerization reaction prevailed. Due to the competition between the depolymerization of GL and the recombination of the intermediates, the decreasing trends of the *M*_w_ and *M*_n_ in the 1:3 mass ratio were higher than those in the 1:1 mass ratio. In addition, the significantly lower values of the *M*_w_ and *M*_n_ of the OGLxxxLs after the 7-d treatment compared to the original GLs in both mass ratios were likely due to the cleavage of ether interunit linkages within lignin macromolecules [6,17,23]. The extent of this cleavage was related to the type of original GLs (source-wood-meal size and PEG molecular mass), as discussed in the subsequent section.

### 2.3. Chemical Structure of Oxidized Glycol Lignins

#### 2.3.1. Thioacidolysis and 2D HSQC NMR Analyses

To verify the changes in OGL structure, analytical thioacidolysis [33,34] and 2D HSQC NMR analyses were conducted on selected OGL samples from the 1:3 mass ratio. The lignin-derived monomers released from the *β*–*O*–4 linkage units following the chemical degradation (thioacidolysis) of the 1-d and 7-d samples of OGL200L, OGl200M, OGL200S, OGL400L, and OGL600L were determined (Table 3). As reported in our previous study [9], the amount of *β*–*O*–4 units in the original GLs varied with the JC wood meal sizes in the following order: GL200L > GL200M > GL200S. After H_2_O_2_ treatment, the *β*–*O*–4 content of the respective OGLs decreased with an increase in treatment time, especially in the OGL200L and OGL200M series. In addition, the amount of *β*–O–4 units in original GLs also varied with the PEG molecular mass (GL200L > GL400L > GL600L) [9]. As observed for OGL200L13, the *β*–*O*–4 content in OGL400L13 and OGL600L13 also considerably decreased after the 7-d treatment. However, the extent of the *β*–*O*–4 decrease became less prominent as the *β*–*O*–4 content in the original GLs decreased, i.e., GL200L (ca. 63% reduction from 211 to 79 μmol/g per OGL) > GL400L (ca. 37% reduction from 146 to 92 μmol/g per OGL) > GL600L (ca. 17% reduction from 100 to 83 μmol/g per OGL) (Table 3). Collectively, these data indicated that the reduction of the *β*–*O*–4 units in the H_2_O_2_-mediated oxidation treatment much depends on the amount of the *β*–*O*–4 units in the original GLs.

**Figure 4:**
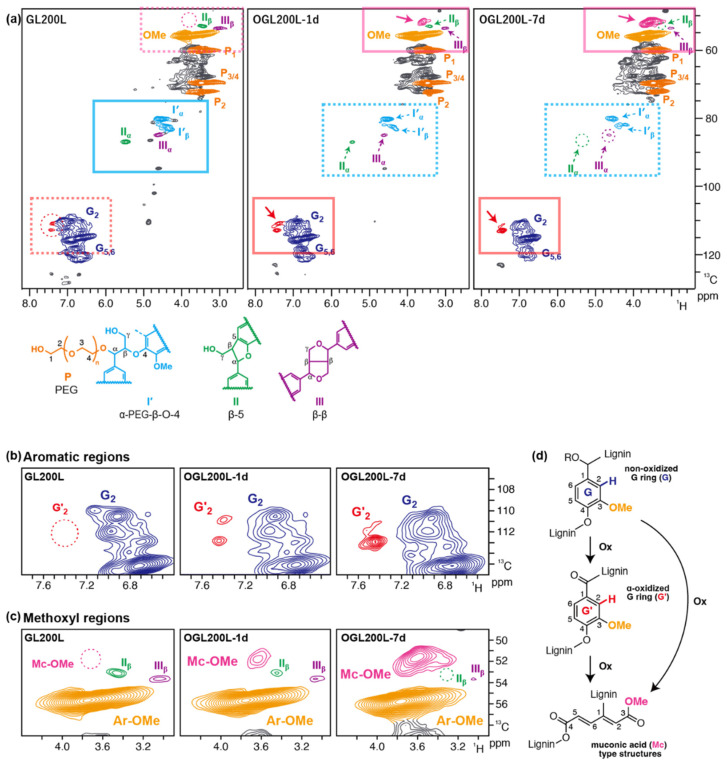
(**a**) 2D HSQC NMR spectra of GL200L and the corresponding OGLs, OGL200L13-1d and OGL200L13-7d. (**b**) Inset of aromatic and (**c**) methoxy regions. (**d**) Ring-opening reaction of guaiacyl aromatic ring into muconic acid (Mc)-type structures.

Selected OGL200 samples were subjected to further in-depth structural analysis using 2D HSQC NMR (Figure 4 and Table 4). As reported in our previous study [9], PEGylation primarily occurred in *α*-benzyl carbon during the acid-catalyzed PEG solvolysis of JC wood meals although PEGylations in other lignin hydroxyl groups are also plausible albeit without clear demonstrations by NMR. In this reaction, almost all *α*-OH-*β*–*O*–4 linkage units in the JC wood meal lignin were converted into *α*-PEG-*β*–*O*–4 linkage units (**I’**) in the resulting GLs, along with the *β*–5 (**II**) and *β*–*β* (**III**) linkage units mostly intact (Figure 4a). After H_2_O_2_ treatment, the signals from these three major linkage units decreased with an increase in treatment time. This result indicated partial degradation of these major linkage types including *β*–*O*–4 linkages during the H_2_O_2_ treatment and was consistent with the thioacidolysis data as shown in Table 3. Notably, the *β*–5 signals (**II**) were almost undetected in all the OGL samples with the 7-d treatment. This result suggests that *β*–5 linkages were degraded more preferentially over *β*–*O*–4 and *β*–*β* linkages, and/or that the solubilized low-molecular-mass fractions after the H_2_O_2_ treatment contained substantially more *β*–5 units than the remaining high-molecular-mass solid fractions which were subjected to the current NMR analysis. Simultaneously, clear aromatic signals from the *α*-oxidized guaiacyl ring (**G’**) (Figure 4a,b) and new methoxy signals (**Mc-OMe**) (Figure 4a,c) corresponding to aliphatic methoxy groups in muconic acid (Mc)-type structures (Figure 4d) apart from those of the normal aryl methoxy groups in guaiacyl rings (**Ar-OMe**) were detected in the HSQC spectra of all the OGL samples tested in this study [35,36].

The contour integration data of the HSQC NMR spectra were used to roughly compare the degrees of oxidation between the OGL samples. The contour integration data (Table 4) suggested that the formation of these oxidized structures was facilitated as the treatment time increased. The formation of the Mc-type structures was clearly more apparent in OGL200L (**Mc-OMe**/**Ar-OMe**, 0.12 after 7-d treatment) than in OGL200M and OGL200S (**Mc-OMe**/**Ar-OMe**, 0.03 and 0.01, respectively, after 7-d treatment). Meanwhile, the extent of the formation of the *α*-oxidized guaiacyl ring was also more apparent in OGL200L (**G’**/**G**, 0.30 after 7-d treatment) than in OGL200M and OGL200S (**G’**/**G**, 0.23 and 0.19, respectively, after 7-d treatment). These results indicate that, as consistent with the thioacidolysis data, the extent of the room-temperature H_2_O_2_-mediated oxidation reaction of the lignin backbone structures is much dependent on the original GL structures.

Treating lignin derivatives with oxidative reagents, including oxygen, H_2_O_2_, ozone, and peroxy acid, can break down the majority of ether linkages (e.g., *β*–*O*–4 linkages) and a portion of C-C linkages through different mechanisms [6,15,21]. Additionally, Mc-type structures can be detected through aromatic ring cleavage using 2D HSQC analysis if lignin oxidation occurs under acidic or neutral conditions [35,37]. As reported by Ma et al. [27], the oxidative depolymerization of a lignin model compound through a mild Fenton reaction mechanism using a combination of chalcopyrite (CuFeS2) catalysts with H_2_O_2_ in acetate buffer (pH = 2–4) produced DCAs, such as succinic, malonic, and malic acids, due to a ring-opening reaction. The results suggested that HO^+^ and HO˙ were present as the primary reactive species, where HO^+^ is proposed to be responsible for the depolymerization of lignin to monomeric phenolic compounds. Meanwhile, HO˙ is necessary for aromatic ring cleavage and DCA formation. Monomeric phenolics, benzoquinone, and Mc derivatives were suggested as the key intermediates. In this study, the H_2_O_2_-mediated oxidation of GL200L at room temperature could follow the similar mechanism, such as the *β*–*O*–4 and *β*–5 cleavages and the formation of Mc-type structures through aromatic ring cleavage as shown by the thioacidolysis and 2D NMR analyses. These data collectively suggest that the chemical structure of GLs, particularly GLxxxL containing abundant *β*–*O*–4 linkage units, are susceptible to mild H_2_O_2_-mediated oxidative treatment.

#### 2.3.2. Attenuated Total Reflectance-Fourier Transform Infrared (ATR-FTIR) Analysis

The effects of source-wood-meal size (GL200L, GL200M, and GL200S) and PEG molecular mass (Gl200L, GL400L, GL600L) on the structural changes of oxidized OGLs (OGL200L, OGL200M, OGL200S, OGL400L, and OGL600L) with the H_2_O_2_ treatment time were further evaluated using ATR-FTIR spectra analysis. Although ATR-FTIR is a qualitative method, a semiquantitative determination method was used to calculate the relative absorbance intensity (RI) of the selected characteristic absorption bands using the absorption intensity of 1269 cm^−1^ (corresponding to G ring breathing) as an internal reference to normalize all spectra after the ATR correction and baseline adjustment.

**Figure 5:**
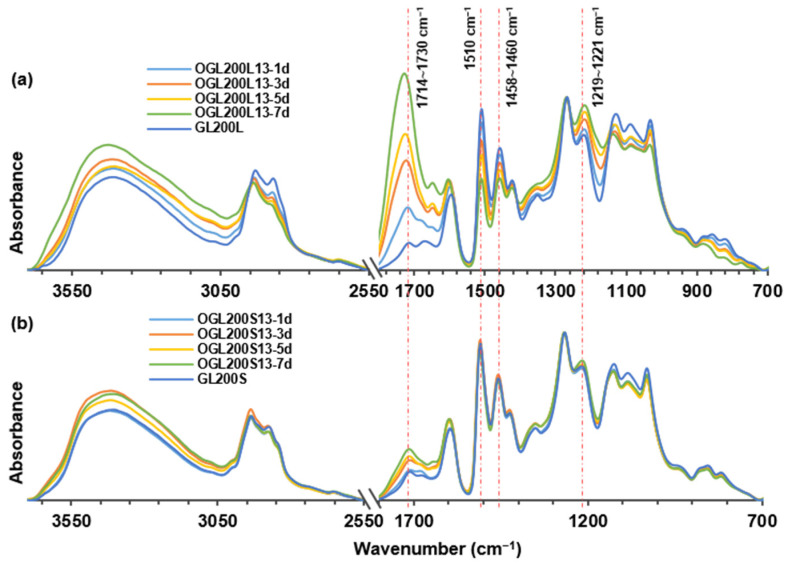
ATR-FTIR spectra of OGLs: (**a**) OGL200L13 series and (**b**) OGL200S13 series as a function of H_2_O_2_ treatment time.

As shown in Figure 5, the typical ATR-FTIR spectra of control GL200L and GL200S showed incorporated PEGs at 2873–2875 cm^−1^, 1350 cm^−1^, 1124–1132 cm^−1^, and 941–949 cm^−1^ corresponding to C-H stretching, -CH_2_ wagging (out-of-plane bending), C-O stretching, and -CH_2_ rocking (in-plane bending) vibrations, respectively. The spectra also showed characteristic absorption bands of lignin macromolecules, such as 1597–1599 cm^−1^ (aromatic ring skeletal vibration and C=O stretching), 1510–1512 cm^−1^ (aromatic ring skeletal vibration), 1458–1460 cm^−1^ (C-H deformation in -CH_2_ and -CH_3_), 1423–1427 cm^−1^ (aromatic skeletal vibration combined with C-H in-plane deformation), 1269–1271 cm^−1^ (G ring breathing with C=O stretching), 1221–1225 cm^−1^ (C-C, C-O, and C=O stretching), 1124–1132 cm^−1^ (C-O stretching in an aromatic-aliphatic ether) and 1032–1034 cm^−1^ (aromatic C-H in-plane deformation) [11,38]. The IR spectra of the OGL200L13 series (Figure 5a) showed considerable structural changes compared to the original GL200L. Conversely, a minor change occurred in the OGL200S13 series (Figure 5b) compared to GL200S, consistent with the thioacidolysis data (Table 3).

**Figure 6:**
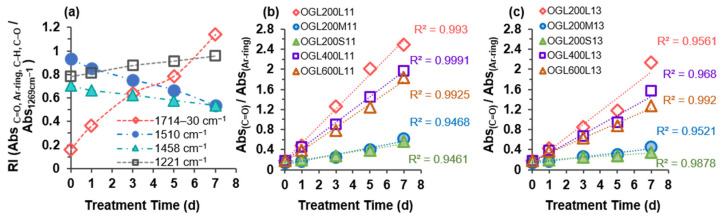
(**a**) Relative absorbance intensity (RI) of carbonyl (C=O) stretching; aromatic ring skeletal vibrations; C-H deformation in -CH_2_ and -CH_3_; and C-C, C-O, and C=O stretching of OGL200L13. (**b**) Absorbance ratio of carbonyl stretching and aromatic ring skeletal vibration of all OGL11 series and (**c**) OGL13 series as a function of H_2_O_2_ treatment time.

Generally, the IR spectra of both OGLs series showed a broadening of the hydroxyl stretching band, with increasing intensity at 3404–3435 cm^−1^, corresponding to the hydroxyl stretching vibration of phenolic and aliphatic groups, as well as additional formation from new carboxy groups. Particularly, upon H_2_O_2_-mediated oxidation treatment, there were four major spectral changes in the 1800–1000 cm^−1^ regions of the OGL200L13 (also 11) series compared to those of original GL200L, which displayed absorption bands at 1714–1730 cm^−1^, 1510 cm^−1^, 1458–1460 cm^−1^, and 1219–1221 cm^−1^ (Figure 5a and Figure 6a). There was a significant change in the increasing spectral intensities at 1714–1728 cm^−1^ associated with the C=O stretching in unconjugated ketones and conjugated acids/esters [11] with an increase in treatment time. This phenomenon implied that the room-temperature H_2_O_2_-mediated oxidation of GL in an acidic medium generated partially oxidized GLs with additional carbonyl functional groups, such as carboxy groups. A gradual increase in C-C, C-O, and C=O stretching at 1219–1221 cm^−1^ further supported the increasing trend of carboxy functional groups. Simultaneously, an apparent reduction in the absorption intensity at 1510 cm^−1^ (aromatic ring skeletal vibrations) and 1458–1460 cm^−1^ (C-H deformation in -CH_2_ and -CH_3_) proved the partial ring cleavage of the G ring (Figure 6a).

Consistent with the results of the 2D HSQC NMR analysis, the partial aromatic ring-opening produces OGLs containing carboxy groups (i.e., muconic acid-type structures). This phenomenon is strongly supported by an increase in the IR spectral intensity of C=O stretching at the expense of a reduction in aromatic ring skeletal (C-C, C=C, and C-H) vibrations. The semiquantitative evidence for the degradation of aromatic structures with an increase in treatment time is displayed in Figure 6b,c by calculating the ratio between the relative absorbance intensities of C=O stretching (1714–1730 cm^−1^) and aromatic ring skeletal vibrations (1510 cm^−1^) for all the OGLs series from both mass ratios. Formation of carboxy groups through partial aromatic ring cleavage was accelerated linearly with an increase in treatment time in all OGL series, where the rate of ring cleavage reaction in the OGLxxxL11 and OGLxxxL13 series was higher than that of the OGL200/M/S-11 and OGL200/M/S-13 series. The optimal fit of treatment-time-dependent oxidative ring cleavage was observed in the OGLxxxL-11 series with R^2^ = 0.99 and OGLxxxL-13 series with R^2^ = 0.96–0.99 (Figure 6b,c).

The overall chemical structure analyses revealed that the original GL prepared from the PEG 200 solvolysis of the source-wood-meal size of L, GL200L, underwent the maximum degradation (with the maximum mass loss shown in Figure 2) during room-temperature H_2_O_2_-mediated oxidation treatment and subsequently produced the partially oxidized products (OGL200Ls) with the maximum number of carboxy functional groups. Since the GL200L sample has the maximum content of *β*–*O*–4 linkage units, the maximum reactivity of GL200L in this study revealed a strong structure-property relationship between the retained native lignin structure (i.e., the relative abundance of *β*–*O*–4 linkage content) and oxidative reactivity.

#### 2.3.3. Determination of Carboxy Group Content Using Potentiometric Titration

The GL solution exhibits a certain color (red-brown to light-brown) when the powder samples are dissolved in an aqueous dioxane solution (dioxane: H_2_O_2_-1:1, *v*/*v*). Potentiometric titration [39,40] was performed to determine the carboxy-containing entities of OGLs due to H_2_O_2_-mediated oxidation treatment. Quantifying carboxy group content shows the degree to which aromatic structures have been degraded or OGLs have been upgraded with carboxy functional groups. The carboxy group content increased with an increase in treatment time in all OGL series, depending on the nature of original GLs prepared from source-wood-meal sizes (OGL200L > OGL200M > OGL200S) and PEG molecular masses (OGL200L > OGL400L > OGL600L) (Table 5).

Collectively, the carboxy group content of all OGL series is in the following order: OGL200L > OGL400L > OGL600L > OGL200M > OGL200S. For example, for the highest-reactive OGL200L sample, the value was increased in the 1.75 mmol/g sample (1:1 mass ratio) and 1.63 mmol/g sample (1:3 mass ratio) through a 7-d treatment compared to its initial value (GL200L: 0.29 mmol/g sample). Meanwhile, the least-reactive sample of OGL200S exhibited the minimum carboxy group content, where the value was increased in the 0.55 mmol/g sample (1:1 mass ratio) and 0.27 mmol/g sample (1:3 mass ratio) after the 7-d treatment. The initial value of the GL200S was 0.26 mmol/g.

He et al. [17] reported that oxidized kraft lignin with a carboxylate group content of 1.53 mmol/g was obtained under the optimal oxidation conditions of 80 °C for 2 h, with a 0.77 NaOH/H_2_O_2_ molar ratio and 2.85 H_2_O_2_/lignin molar ratio. Compared with the initial value of kraft lignin (1.01 mmol/g), the carboxylate group content in the oxidized kraft lignin was increased by 0.52 mmol/g through H_2_O_2_ treatment under alkaline conditions. The treatment condition and initial lignin derivative used for H_2_O_2_-mediated oxidation treatment differ completely; thus, it is hard to compare them. However, the value obtained from the optimum oxidation condition in He et al. [17] is similar to the value obtained from the least-reactive sample, OGL200S11-7d. The results revealed that the structural feature of the original/initial lignin derivative sample is crucial to upgrading insoluble oxidized lignin derivatives with carboxy functionalization through H_2_O_2_-mediated oxidation treatment. Kraft lignin was processed from byproducts of kraft pulping, whereas the GL used in this study was processed directly from softwood-meal as the main product. Although both lignin derivatives were classified as technical lignins, the structural features of GL can be controlled substantially by varying the source-wood-meal size and PEG molecular mass. Consequently, GLs with abundant native lignin structure (*β*–*O*–4 linkage units), such as the GL200L in this study, are easy to upgrade, and thus, a considerable amount of carboxy functionalization was achieved through room-temperature H_2_O_2_-mediated oxidation treatment.

**Figure 7:**
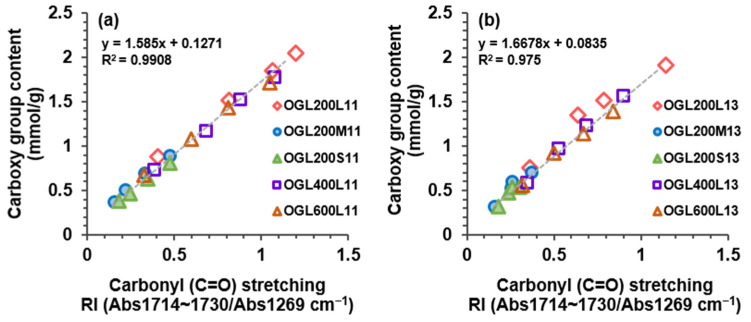
Correlation between the values of carboxy group content determined through potentiometric titration and RI values of the carbonyl absorption band of (**a**) OGL11 and (**b**) OGL13 series through the room-temperature H_2_O_2_-mediated oxidation treatment.

Notably, the increasing trends of carboxy group content with an increase in the treatment time were found to be consistent with the trends of the RI values of the C=O stretching band at 1714–1730 cm^−1^ from the ATR-FTIR spectral analysis (Section 2.3.2). Although the ATR-FTIR analysis is a qualitative method, the RI values of the normalized ATR-FTIR absorption band exhibited a strong correlation with the values quantified through potentiometric titration. Figure 7 shows that, compared to the values of the RI (C=O stretching), the carboxy group content determined through potentiometric titration displayed a strong correlation value of R^2^ = 0.991 and 0.975 for 1:1 and 1:3 mass ratios, respectively. Due to the simplicity of the ATR-FTIR sampling technique, which directly, quickly, and simply measures various sample types without additional preparation, the carboxy group content can be easily estimated using this correlation.

### 2.4. Thermal Properties of Oxidized Glycol Lignins

The thermal properties of the five original GLs and 40 OGL samples were determined using thermomechanical analysis (TMA) and thermogravimetric analysis (TGA) thermal analyzers. TMA determines the glass transition temperature (*T*_g_) and viscous thermal flow temperature (*T*_f_). Meanwhile, TGA determines the decomposition temperatures (*T*_d_), such as decomposition-starting temperature (*T*_dst_) and maximum decomposition temperature (*T*_dmax_), which are temperatures corresponding to a 5% weight loss and maximum weight loss, respectively. Unlike other known technical lignins, GLs used in this study are PEG-modified lignin derivatives, and hence, most of the control GLs exhibit *T*_f_ in addition to *T*_g_ under 200 °C [9,11].

**Figure 8:**
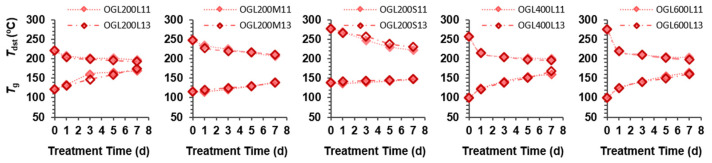
Thermal properties, including *T*_g_ and *T*_dst_, of OGLs as a function of H_2_O_2_ treatment time.

Notably, *T*_f_ was absent in all OGLs series after H_2_O_2_ treatment. However, the presence of PEG chains was confirmed through the 2D HSQC NMR analysis (Figure 4a). During oxidative treatment, the partial liberation of PEG moieties, formation of crosslinked PEG chains, and recombination of the intermediates can occur. Thus, the limited mobility of PEG chains in OGLs cannot allow them to proceed to the viscous thermal flow phase, as in original GLs. Instead, *T*_g_ increased with an increase in treatment time, the extent of which depended on the type of original GL. Consequently, OGLs with various *T*_g_ values ranging from 115 °C to 175 °C with varying degrees of carboxy functionalization were obtained. Both mass ratios displayed a similar trend in all OGL series (Figure 8).

Figure 8 shows the thermal stability (*T*_dst_) of OGLs determined through TGA analysis. In contrast to *T*_g_, the values of *T*_dst_ in each OGL series decreased slightly compared to the corresponding original GL and gradually decreased with an increase in treatment time over the temperature range of 193 °C to 268 °C due to the increasing degree of carboxylation. Among the OGL series, *T*_dst_ was determined in the following order: OGL200S > OGL200M > OGL600L > OGL400L > OGL200L series. Meanwhile, the values of *T*_dmax_ (351 °C–369 °C) and the char residues at 800 °C (30–38%) of OGLs exhibited a minor change with an increase in treatment time compared to the corresponding original GL.

## 3. Materials and Methods

### 3.1. Room-Temperature Hydrogen Peroxide Treatment

The five GL samples (Table 1) obtained from the GL production test plant (Forestry and Forest Products Research Institute, Tsukuba, Japan) were used for the H_2_O_2_ treatment. A detailed description of GL production through acid-catalyzed PEG solvolysis of softwood JC was reported in our previous study [9]. GL samples were suspended in a 30 wt.% aqueous H_2_O_2_ solution in 100 mL beaker while stirring to obtain good dispersion and covered with aluminum foil. The mass ratio of H_2_O_2_ to GL was kept as 1:1 and 1:3. After continuous stirring for 6 h, the reaction slurries were kept standing to undergo mild oxidation for 1, 3, 5, and 7 d at room temperature. Forty partially oxidized solid GLs (four samples per one series for 2 mixing ratios), OGLs, were obtained through vacuum filtration. Subsequently, they were rinsed with distilled water and air-dried for 1 d. Finally, the air-dried OGLs were subjected to vacuum drying for 2 d to determine the product yield (Equation (3)), and further analyses were conducted. The pH of the filtrate was found to be in the range of pH 2 to 3. However, the filtrate was not investigated further in this study.

(3) $$OGL\;\mathrm{yield}\;(\%)=\frac{OGL}{GL}\times 100,$$

where, *OGL* and *GL* represent the partially oxidized glycol lignin obtained through room- temperature H_2_O_2_ treatment and the original sample, respectively.

### 3.2. Chemical Analyses

Klason lignin assay [9] and analytical thioacidolysis [33,34] were performed as previously described, and the average values of duplicate or triplicate runs were reported.

The carboxy group content of OGLs was quantitatively determined through potentiometric titration using a Metrohm 916 Ti-Touch equipped with a magnetic stirrer (Metrohm Japan Ltd., Tokyo, Japan) as reported by Dence [39,40] with modifications. An aqueous solution of 0.1 M NH_4_OH (known factor) and p-hydroxybenzoic acid were used as the titrant and the internal standard, respectively. The dioxane/water (1:1, *v*/*v*) solution was used for the dissolution of GLs, OGLs, and the internal standard. The sample solution was prepared by dissolving GLs and OGLs (~0.12 g based on the Klason lignin content) in 50 mL of aqueous dioxane solution, followed by the addition of 5 mL internal standard solution and 1 mL of 0.5 M HCl (known factor). Subsequently, the sample solution was shaken gently for 5 min to obtain complete mixing into one phase and titrated with 0.1 M NH_4_OH. The carboxy group content was calculated with the following Equation (4):

(4) $$\mathrm{mmol}\;\mathrm{RCOOH}\;\mathrm{per}\;g\;\mathrm{sample}=\frac{\left(b-a\right)\times \left[NH_{4}OH\right]-c}{w},$$

where *a* and *b* are the titrant volumes (0.1 M NH_4_OH) at end points 1 and 2, [NH_4_OH] is the NH_4_OH concentration corrected for HCl interference, *c* is the mmol of the internal standard (p-hydroxybenzoic acid), and *w* is the weight of the analyzed GLs and OGLs.

### 3.3. Spectral Analyses

2D HSQC NMR spectra were acquired on a Bruker Biospin AVANCE III 800US spectrometer (Bruker Biospin, Billerica, MA, USA) equipped with a cryogenically cooled 5 mm TCI gradient probe. Approximately 15 mg vacuum-dried fine powder sample was dissolved in 600 μL of dimethysulfoxide (DMSO)-*d*_6_ for NMR analysis; all the GL and OGL samples tested in the current study were soluble in DMSO-*d*_6_. Adiabatic 2D HSQC experiments were conducted using the standard Bruker implementation (“hsqcetgpsp.3”) and previously described acquisition parameters [9,41]. Data were processed and analyzed using Topspin 4.0 software (Mac; Bruker Biospin, Billerica, MA, USA) as previously described [9,41]. The central DMSO solvent peaks (δ_C_/δ_H_: 39.5/2.49 ppm) were used as an internal reference. For contour integration analysis, well-resolved **G_2_**, **G’_2_**, **Ar-OMe**, and **Mc-OMe** contours (Figure 4) were integrated.

FTIR spectrometer (Nicolet is50, Thermo Fisher Scientific, Madison, WI, USA) equipped with a single-crystal diamond top plate ATR accessory was used to collect the ATR-FTIR spectra over a range of 4000 to 400 cm^−1^ with 32 scans and a 4 cm^−1^ resolution.

### 3.4. Size Exclusion Chromatography (SEC)

The weight average and number average molecular mass were determined using a Shimadzu Prominence LC-20AD (Shimadzu Corporation, Kyoto, Japan) system equipped with a two-column sequence of Shodex KD-802 and KD-804. Refractive index and UV (280 nm) detectors were used to monitor the column effluent. Fluka poly(ethylene glycol)/poly(ethylene oxide) standard ReadyCal sets (Sigma-Aldrich Japan G. K., Tokyo, Japan) were used for the molecular mass calibration. The standard, GL, and OGL samples were dissolved in the eluent (10 mM LiBr dissolved in *N*, *N*-dimethylformamide) and filtered through a 0.20-μm poly(tetrafluoroethylene) syringe filter (ADVENTEC) before being set into an autosampler unit. The eluent flow rate and the column oven temperature were 1.0 mLmin^−1^ and 40 °C, respectively.

### 3.5. Thermal Analyses

TMA was performed to determine the glass transition temperature (*T*_g_) and the viscous thermal flow temperature (*T*_f_) using Q400 TMA (TA Instruments-Waters LLC, New Castle, DE, USA). The sample assembly comprised a platinum sample pan (ϕ 6 × 2.5 mm^2^) loaded with a vacuum-dried fine powder sample (7–8 mg) and an aluminum top cover (ϕ 4 mm). After setting the sample assembly between a quartz stage and a movable probe, the sample was heated from room temperature to 300 °C at a rate of 5 °C min^−1^ under a nitrogen environment (100 mL min^−1^) and an applied load of 0.05 N [9,10,11].

TGA was performed to determine the thermal *T*_dst_ and *T*_dmax_ using Q500TGA (TA Instruments-Water LLC, New Castle, DE, USA). A platinum pan (100 μL) loaded with a vacuum-dried fine powder sample (7–8 mg) was initially heated to 105 °C at a rate of 10 °C min^−1^ and held at 105 °C for 20 min. Subsequently, it was heated to 850 °C at a rate of 10 °C min^−1^ under a nitrogen flow (60 mL min^−1^ for the sample compartment and 40 mL min^−1^ for the balance compartment).

The duplicate data sets were analyzed using TA universal analysis software, where *T*_g_ and *T*_f_ are defined as onset temperature, as well as *T*_dst_ and *T*_dmax_ are defined as temperatures at 5% weight loss and maximum weight loss, respectively. *T*_dst_ was recalculated after the weight stabilized at 105 °C.

## 4. Conclusions

In this study, softwood-derived PEG-modified GLs, prepared from various JC wood meal sizes through an acid-catalyzed PEG solvolysis, were further upgraded using room-temperature oxidative treatment with H_2_O_2_ as a green oxidant. Although the exact reaction mechanism has not been verified, the 2D HSQC NMR and thioacidolysis data demonstrated that oxidative functionalization of GLs involved cleavage of *β*–*O*–4 linkages and the ring-opening reaction of a partially α-oxidized guaiacyl ring to form muconic acid-type structures. Notably, oxidation reactivity is strongly correlated with the amount of the retained native lignin structure (abundant *β*–*O*–4 linkages) present in the original GLs. Accordingly, the original GL200L, which has an abundant native lignin structure, was easy to upgrade to carboxy-rich OGL (2.04 mmol/g) in the corresponding OGL200L11-7d. Conversely, GL200S, with an inferior amount of *β*–*O*–4 linkage units, was upgraded with a 0.81 mmol/g carboxy group content in the corresponding OGL200S11-7d. Consequently, reduced and increased OGL yields were obtained for the OGL200L series and OGL200S series, respectively. In addition, the amount of carboxy content determined by potentiometric titration had a high correlation (R^2^ = 0.975–0.991) with the values of relative absorbance intensity of carbonyl stretching vibration from ATR-FTIR analysis. This correlation will enable the easy estimation of OGL carboxy group content through simple ATR-FTIR analysis in future studies on large-scale GL upgrading. The carboxy-rich OGLs in this study can be directly used as green dispersants, adsorbents, and binders and as bio-based substitutes for fossil-based polymers in material syntheses, such as epoxy resin synthesis. Further investigation in regard to manipulating the mixing ratios and/or reaction temperatures to reduce H_2_O_2_ is necessary to improve the cost efficiency and sustainability of the upgrading process.

## Figures and Tables

**Table 1:** GL samples and corresponding OGLs samples.

Original GL			OGL		
Raw Materials		GL	Mass Ratio H_2_O_2_/GL, 1:1	Mass Ratio H_2_O_2_/GL, 1:3	Treatment Time (d)
JC Size	PEG				
JC-L	200	GL200L	OGL200L11	OGL200L13	1, 3, 5, 7
JC-M	200	GL200M	OGL200M11	OGL200M13	1, 3, 5, 7
JC-S	200	GL200S	OGL200S11	OGL200S13	1, 3, 5, 7
JC-L	400	GL400L	OGL400L11	OGL400L13	1, 3, 5, 7
JC-L	600	GL600L	OGL600L11	OGL600L13	1, 3, 5, 7

**Table 2:** Klason lignin and acid soluble-lignin content of OGLs.

OGLs	Original GL (%)	1 d		3 d		5 d		7 d	
		H_2_O_2_/GL ^1^		H_2_O_2_/GL ^1^		H_2_O_2_/GL ^1^		H_2_O_2_/GL ^1^	
		1:1	1:3	1:1	1:3	1:1	1:3	1:1	1:3
Klason Lignin (%)									
200L	81.3	81.3	81.2	74.1	78.6	70.5	75.2	66.6	66.0
200M	81.4	81.8	81.4	81.5	81.7	79.8	79.7	79.9	80.4
200S	85.1	84.8	84.4	84.5	85.0	84.5	84.3	83.7	85.2
400L	76.7	81.4	79.1	78.9	78.7	71.9	76.3	66.7	72.2
600L	75.4	80.2	79.0	80.1	79.2	75.0	78.7	70.2	74.2
Acid Soluble Lignin (%)									
200L	0.5	2.0	2.1	6.8	4.8	10.6	6.9	12.7	11.8
200M	0.5	0.8	0.8	1.3	1.2	1.8	1.6	2.5	2.3
200S	0.4	0.7	0.8	1.0	1.0	1.5	1.3	2.1	1.6
400L	0.5	1.4	1.4	3.2	3.1	7.1	4.7	10.4	6.9
600L	0.6	1.4	1.6	2.7	3.0	5.3	3.4	8.7	6.4

^1^ H_2_O_2_/GL mass ratio.

**Table 3:** Yields of *β*–*O*–4 derived monomeric compounds through thioacidolysis of OGLs.

	Original GL (μmol/g)	1 d (μmol/g)	7 d (μmol/g)
OGLs	Per GL	Per OGL	Per OGL
OGL200L13	211 ± 8.7	175 ± 11.3	79 ± 6.8
OGL200M13	145 ± 1.9	115 ± 2.6	114 ± 4.6
OGL200S13	23 ± 1	19 ± 0.5	21 ± 1.2
OGL400L13	146 ± 9.4	149 ± 12.1	92 ± 14.7
OGL600L13	100 ± 4.2	110 ± 16.2	83 ± 9.0

**Table 4:** Rough estimations of oxidation degree of OGLs based on HSQC NMR contour integration.

	Original GL		1 d		7 d	
OGLs	G’/G	Mc-OMe/Ar-OMe	G’/G	Mc-OMe/Ar-OMe	G’/G	Mc-OMe/Ar-OMe
OGL200L13	0.06	0	0.22	0.02	0.30	0.12
OGL200M13	0.04	0	0.14	0.01	0.23	0.03
OGL200S13	0.07	0	0.10	trace	0.19	0.01

**G’**, *α*-oxidized guaiacyl ring. **G**, non-oxidized guaiacyl ring. **Mc-OMe**, aliphatic methoxy groups in muconic acid-type structures derived from ring-opening reactions of guaiacyl ring. **Ar-OMe**, aryl methoxy groups in guaiacyl ring.

**Table 5:** Carboxy group content of OGLs determined through potentiometric titration.

OGLs	Original GL mmol/g	1 d (mmol/g)		3 d (mmol/g)		5 d (mmol/g)		7 d (mmol/g)	
		H_2_O_2_/GL ^1^		H_2_O_2_/GL ^1^		H_2_O_2_/GL ^1^		H_2_O_2_/GL ^1^	
		1:1	1:3	1:1	1:3	1:1	1:3	1:1	1:3
200L	0.29	0.88	0.76	1.52	1.35	1.85	1.51	2.04	1.92
200M	0.25	0.37	0.32	0.50	0.53	0.69	0.60	0.89	0.71
200S	0.26	0.37	0.31	0.46	0.48	0.63	0.54	0.81	0.53
400L	0.25	0.74	0.59	1.18	0.97	1.53	1.24	1.78	1.57
600L	0.26	0.67	0.55	1.08	0.92	1.43	1.14	1.72	1.39

^1^ H_2_O_2_/GL mass ratio.
